# Implementation Evaluation of a Teledermatology Virtual Clinic at an Academic Medical Center

**DOI:** 10.21203/rs.3.rs-2558425/v1

**Published:** 2023-02-28

**Authors:** Meenal Kheterpal, Ethan D. Borre, Matilda W. Nicholas, Edward W. Cooner, Donna Phinney, Kelly Gagnon, Leah L. Zullig, Heather A. King, Elizabeth J. Malcolm, Suephy C. Chen

**Affiliations:** Department of Dermatology, Duke University; DUMC: Duke University School of Medicine; DUMC: Duke University School of Medicine; DUMC: Duke University School of Medicine; DUHS: Dow University of Health Sciences; DUHS: Dow University of Health Sciences; DUMC: Dongguk University Medical Center; DUMC: Duke University School of Medicine; DUMC: Duke University School of Medicine; DUMC: Duke University School of Medicine

**Keywords:** teledermatology, implementation science, barriers and facilitators

## Abstract

**Background:**

Teledermatology (TD) is an evidence-based practice that may increase access to dermatologic care. We sought to evaluate implementation of TD at four Duke primary care practices.

**Methods:**

We implemented a hybrid TD program where trained primary care providers (PCPs) sent referrals with clinical and dermatoscopic images to dermatology. Patients were seen by dermatologists over video visit within days, and dermatologists managed the patient plan. We evaluated implementation using the Reach, Efficacy, Adoption, Implementation, and Maintenance (RE-AIM) framework using electronic health record data. Implementation barriers and facilitators were collected through surveys (n = 24 PCPs, n = 10 dermatologists, n = 10 dermatology residents).

**Results:**

At four PCP clinics throughout 9/1/2021-4/30/2022 there were 218 TD referrals. Video visits occurred on average 7.5 days after referral and 18/18 patients completing the post-visit survey were satisfied. Adoption varied between clinics, with one placing 22% of all dermatology referrals as TD and another placing 2%. The primary PCP barriers to TD were time burdens, lack of fit in clinic flow, and discomfort with image taking. Top-endorsed potential facilitating interventions included allowing for rash referrals without dermoscopy and assurance for clinical evaluation within 3 days.

**Conclusions:**

Addressing TD process fit into PCP clinic flow and reducing time burdens may increase PCP uptake of TD.

## Introduction

Limited patient access to dermatologic care remains a problem across many regions in the United States, and teledermatology (TD) has shown promise in reducing substantial patient wait times while achieving similar patient outcomes and satisfaction to in-person care.^1–7^ Two common formats of TD include 1) store-and-forward TD in which a remote dermatologist reviews patient images and forwards their clinical recommendation to the referring provider; and 2) TD video visits in which dermatologists review images followed by a video visit with the patient to confirm their diagnostic suspicion and directly counsel the patient.^8^ While there has historically been more research around store-and-forward TD, recent Medicare payment changes allowing for reimbursement parity for video and in-person visits due to COVID-19 have popularized the synchronous TD video visit format.^2, 9–11^

**Figure 1:**
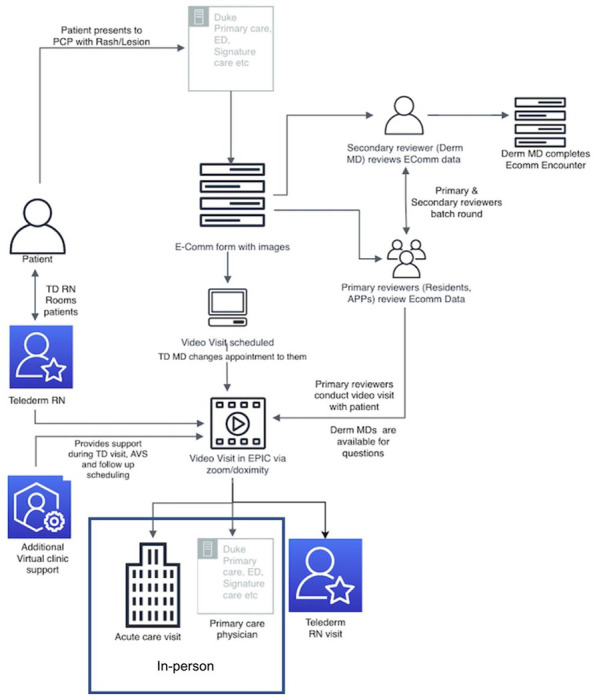
Teledermatology virtual clinic process depiction.

In September 2021, to address patient wait times of > 6 months (for both primary care referrals and new patients), Duke Dermatology implemented a hybrid TD virtual clinic in four Duke Primary Care (DPC) pilot sites. In the hybrid care model, primary care providers (PCPs) were trained to take clinical and dermatoscopic images (Fig. 1). Then then transmitted an e-consult with basic information as well as clinical and dermatoscopic images to dermatology. A video visit between the patient and the TD team was subsequently scheduled to occur at least 3 days after the e-consult. The TD dermatology team consisted of an initial reviewer (dermatology resident or advanced practice provider, APP) who prereviewed the images and charts and then “batch rounded” with the TD attending. Subsequently, the initial reviewer conducted the video visit with the patient; the attending was also on the video when the initial reviewer was a dermatologist resident, but not when they were an APP. While the effectiveness of TD has been previously demonstrated, the barriers and facilitators to its implementation are less well understood.^12–14^ There are numerous complex factors related to synchronous TD’s successful implementation and understanding of optimal implementation conditions will allow for better dissemination of the evidence-based practice.^14–16^

In this study we sought to assess the preliminary implementation outcomes of the TD virtual clinic as well as map barriers and potential facilitators to its implementation. Our findings may provide a guide for other centers considering implementing hybrid TD video visits.

## Methods

We used a quality-improvement study design and followed the Standards for Quality Improvement Reporting Excellence.^17^

### Setting and participants

Duke Dermatology and Duke Primary Care implemented the TD virtual clinic in 4 pilot primary care clinics beginning in September 2021. Participants in this study included dermatologist and PCPs that participated in the TD clinic.

### Intervention

Leadership across Duke Dermatology, Duke Health, and Duke Primary Care collaborated to define the TD virtual clinic process (Fig. 1). Implementation planning was undertaken using the Exploration, Preparation, Implementation, Sustainment (EPIS) framework.^18, 19^ We trained PCPs using an introductory clinical meeting (20 minutes), followed by an optional learning module (20 minutes) to be completed by providers virtually. The training included the description of the process and specialized image capture training, in particular: types of images (forest, close-up, dermoscopy), use of complimentary body parts for rashes, common pitfalls, and examples of excellent and poor images, followed by an image quiz to assess knowledge gaps. Images were taken with an iPad and compatible dermatoscopic and uploaded directly to the electronic health record for transmission to the dermatology reviewing team. All e-consults required clinical and dermatoscopic images. Dermatologists were able to bill video visits to payers.

### Measurement and analysis

#### Implementation framework identification and use

We used electronic health record (EHR) data to measure implementation success consistent with the Reach, Effectiveness, Adoption, Implementation, and Maintenance (RE-AIM) framework.^13, 20^ RE-AIM is an evaluation framework.^20–22^ To complement RE-AIM, we also used the EPIS framework.^18, 22, 23^ As a process model, EPIS provided critical structure for implementation planning and identification of barriers and facilitating factors. We assessed barriers and facilitators of virtual clinic implementation using surveys distributed to PCPs, and dermatology attendings, residents, and APPs. Compiling the survey results, we identified key barriers to our TD process implementation and mapped these barriers to potential implementation strategies.

#### Measuring implementation outcomes

To prioritize implementation outcome collection of the new TD process across the RE-AIM framework, we distributed a survey of previously published potential RE-AIM outcomes to TD leadership (see Table 1 for a list of implementation outcomes; four medical directors of pilot sites and three Duke Dermatology leaders).^13, 20^ We created a TD dashboard to collect real-time implementation outcomes important to the leadership, prioritizing outcomes rated highly by primary care and dermatology leadership (Table 1). The dashboard aggregated TD implementation and patient outcomes from the EHR. Patient satisfaction was measured as a post-video single question that asked whether the patient felt their clinical needs were adequately address (options: strongly agree, agree, disagree, strongly disagree).

#### Identifying barriers and facilitators to TD implementation

**Figure 2:**
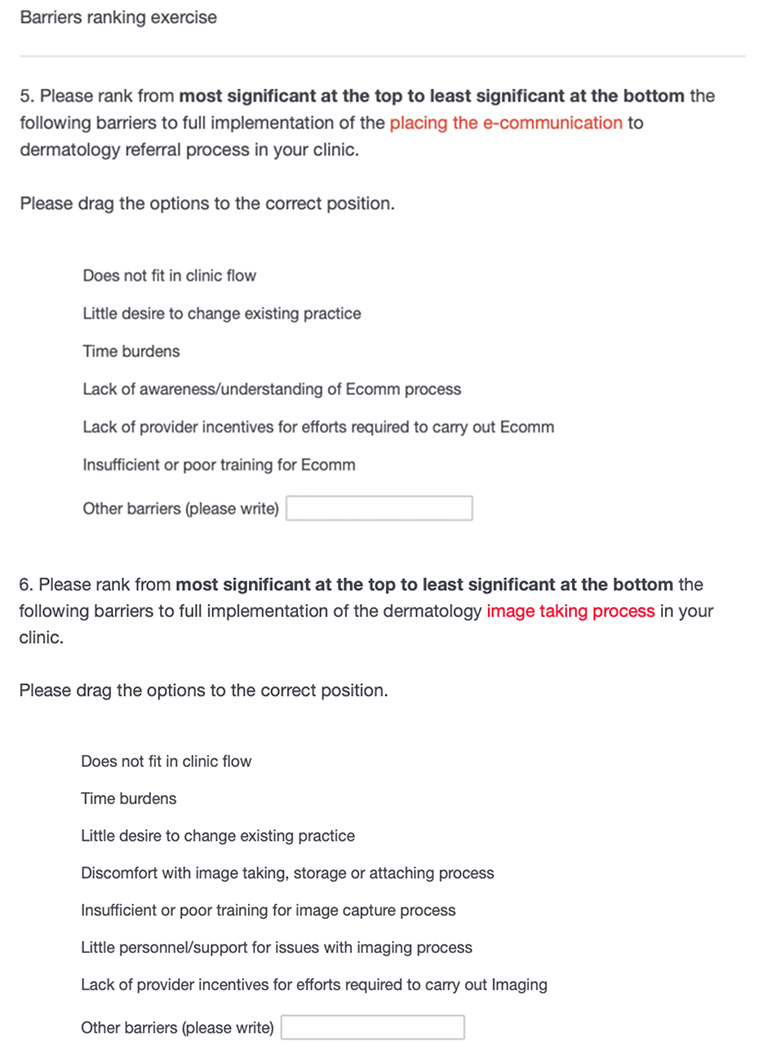
Barriers ranking survey.

To identify barriers important to PCP implementation of the TD virtual clinic and assess the “Implementation” portion of RE-AIM (degree to which program was implemented as planned), we designed a ranking survey of potential barriers to implementation across e-consult placement and image taking (Fig. 2). To identify the barrier list for this survey, we first identified an expanded list of all potential barriers (n = 17) using the EPIS framework, literature review, and dermatologist input. We then selected the most relevant barriers through unstructured discussions with the four pilot site medical directors. The abbreviated survey was sent to 73 providers at the pilot sites six months after TD implementation (Appendix 1).

In the same survey, we proposed potential interventions, informed by the literature, to facilitate TD virtual clinic referral for PCPs.^14^ For each potential facilitator, we asked the respondent to indicate whether they strongly disagreed, disagreed, agreed, or strongly agreed the intervention would facilitate implementation of TD.

We sent surveys, modified for relevance, to 10 attending dermatologists and 15 residents staffing the TD clinic to understand barriers to their implementation of the TD process. For the attendings, we assessed their support of facilitator interventions promoting PCP referral. While APPs will be involved in the TD initial reviewing team, they were not involved in TD during this pilot phase and therefore were not surveyed.

## Results

### Patient and provider reach

During 9/1/2021-4/30/2022 at the four pilot clinics, a total of 218 e-consults were placed (Table 2; 154 lesions and 64 rashes). 171 video visits were completed, and the process involved 6 attending dermatologists, 15/15 resident dermatologists, and 30 primary care providers. Of the 171 completed video visits, 20 (12%) were placed by Clinic A (urban; 8 PCPs), 77 (45%) by Clinic B (urban; 16 PCPs), 70 (41%) by Clinic C (urban; 15 PCPs), and 4 (2%) by Clinic D (urban; 8 PCPs). Of patients completing a video visit, 73% self-reported White race, 18% Black race, and 9% other races. This was similar to self-reported race of ambulatory referral patients to dermatology: 72% White race, 17% Black race, and 12% other races.

### Effectiveness and process measures

Of all e-consults, 85% had a scheduled video visit, with 15% unscheduled secondary to patient declining or unable to be reached. 80% of e-consults were scheduled as video visits with the dermatology team in 3 days or less, and the mean time from e-consult to video visit was 7.5 days (compared to in-person wait-times of 6 months). 8% of scheduled video visits were cancellations or no-shows. The average video visit length was 10 minutes, and 1 (1%) video visit was converted to a telephone visit due to patient technology difficulties. All (18/18) patients who completed the satisfaction survey (all TD patients were surveyed, but few completed the satisfaction survey within the timeframe of this analysis) indicated that their clinical goals were met during the video visit. 65% of video visits required a downstream in-person appointment.

### Adoption

The percentage of all PCPs at participating pilot clinics that placed at least one TD e-consult varied between pilot clinic sites: Clinics B and C had more than 75% of PCPs placing at least one referral, whereas Clinic A had 50% and D had 13%. The percentage of total dermatology referrals (ambulatory in-person + TD) that were TD also varied between clinic sites, ranging between 2% at Clinic D to 22% at Clinic C. In other words, Clinic D, compared to other clinics sent the fewest TD consults.

### Identified PCP barriers to TD referral

**Figure 3:**
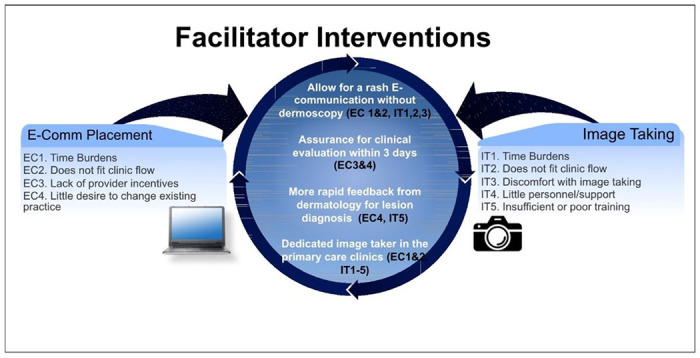
Primary care physician reported barriers to teledermatology e-consult and image taking mapped to proposed facilitating intervention.

Given disparate adoption rates between clinics, and that all clinics had TD referrals comprising ≤ 22% of all dermatology referrals, we assessed barriers to e-consult placement among participating PCPs through the barrier ranking survey. 24 PCPs responded to the survey, which was 31% of the emailed sample. With respect to *e-consult* placement, the highest ranked barriers were time burdens, lack of fit in clinic flow, lack of PCP incentives, and little desire to change existing practice (Fig. 3). For *image taking*, the highest ranked barriers were time burdens, lack of fit in clinic flow, discomfort with image taking, little personnel/support, and insufficient or poor image taking training. Six providers emphasized in free response that dermatoscopic and clinical image taking was the most time-consuming portion of the process, reporting difficulties retrieving the iPad from storage, logging into the EHR, and obtaining images of adequate quality.

### Identified dermatology attending and resident barriers to TD

Eight dermatology attendings responded to the survey (80% response rate). The top three endorsed barriers to TD from the dermatologist perspective were: 1) video technology difficulties on the patient end (75% endorsed as a barrier), 2) concerns with ready availability of in-person follow-up slots for TD patients (63%), and 3) concerns around compensation for TD clinic compared to in-person clinic (38%). Two dermatology attendings (25%) considered image quality to be a concern.

Ten dermatology residents completed the survey (67% response rate), with eight (80%) agreeing that participating in TD is beneficial to their clinical education. The barriers most frequently endorsed by dermatology residents were: 1) concerns with ready availability of in-person follow-up slots (60%), 2) video issues on the patient-end (40%), and 3) video issues on the provider end (40%).

### Potential facilitators of TD referral

Of 10 proposed interventions, those most endorsed were 1) allowing for an e-consult referral for rashes with clinical images only (no dermoscopy), while amenable to perform dermoscopy for lesions; 2) assurance that patients will receive a call to schedule the video visit within 3 days; 3) more rapid dermatologist feedback about lesion diagnosis, and 4) provision of a dedicated image-taker at the primary care clinic (Fig. 3). In general, dermatology attendings agreed with these potential facilitators, ranking allowing for rash referral without dermoscopy and assurance that the patient will be contacted within three days among the top facilitators. We mapped the top-endorsed proposed facilitator interventions to the highest priority barriers in Fig. 3.

## Discussion

The COVID-19 pandemic forced dermatologists to implement TD quickly, with little opportunity for careful evaluation of implementation. Health care systems such as the Veterans Administration have been deploying TD for many years, but these are primarily using the store-and-forward paradigm, which continues to not be reimbursable in scalable manner outside closed health care systems.^24, 25^ A recent systematic review found no existing studies using a comprehensive implementation framework to identify factors influencing teledermatology implementation.^14^ Since then, Peracca et al. recommended the RE-AIM framework to evaluate the VA consultative TD service to rural Veterans.^13^

Our study found that a hybrid TD virtual clinic increased patient access to dermatologic care with high patient satisfaction among the 18 patients who completed the survey. Across RE-AIM, we found strong initial implementation effectiveness (reduced wait times from 6 months to ~ 1 week) but with variable adoption among pilot clinic sites. The reach of the TD program was 200 + patients and there are plans to expand to other clinics. We found that that for PCPs the time required to place the e-consult and take clinical and dermoscopy images were significant barriers to offering TD. Acceptable facilitating interventions to address these barriers included eliminating the dermoscopy requirement for rashes and providing a dedicated image-taker.

There were several unique approaches to our virtual clinic that likely contributed to the early implementation effectiveness. First, there was strong institutional support through a centralized Duke Telemedicine scheduling and support center, that had implemented e-consults for other Duke specialties. The project identified key partners across Duke for financial support, Duke Performance Services for EHR and data aggregation support, and physician champions for operational support and clinical expertise. Importantly, the process included a “virtual clinic” model with TD nurses virtually rooming patients to reduce technology difficulties. This allowed dermatologists to evaluate and treat patients without delays. That said, video technology issues on the patient end, including low quality connections, were endorsed as a barrier by dermatology attendings and residents.^14^ Technological improvements to telemedicine platforms and expanded access to high-speed internet may reduce this barrier in the future.

We present a rigorous application of implementation science frameworks to evaluate TD implementation to provide a guide for other clinical settings. As our TD virtual clinic expands, we will leverage the EHR dashboard and implementation science framework to measure implementation and plan for adaptation. This framework in our setting demonstrated variable PCP adoption of TD at the pilot clinics and we were able to focus our barriers assessment and planned facilitators to address PCP adoption.

Our identified barriers to implementation of a TD virtual clinic may also generalize beyond our four pilot sites. Time burden and clinic fit were among our most frequently cited barriers amongst PCPs, and indeed are commonly recognized barriers to implementation across a wide range of interventions.^26^ Specific to TD, image taking is among the primary and most-time-consuming barriers for PCPs (and image quality for dermatologists). Other TD programs have identified this issue through qualitative research and improved image quality to 99% acceptable images using trained, dedicated image takers (personal communication, SC Chen). Future research might take advantage of existing models of technology adoption in healthcare settings, such as the Fit between Individuals, Task, and Technology framework, to better understand barriers to technology adoption in TD.^27^

We also proposed facilitating interventions, and assessed their preliminary acceptability, to mitigate these barriers. Our decision to allow for rash consultation without dermatoscopic images is supported by recent studies demonstrating low perceived utility of dermatoscopic images for rashes.^28^ We plan to assess this strategy’s effects on implementation outcomes in future studies.

Our study has several limitations. First, we studied a single academic institution, so our findings may not be generalizable to other institutions in other parts of the country. We also did not address the type of hybrid TD where patients send their own photos, without the PCP; we now offer this service but do not yet have enough data to analyze. Second, 76% of PCPs who were administered the TD barriers survey did not respond and for confidentiality reasons we did not collect their clinic affiliation. However, the consistency of top-ranked barriers among all clinicians who did respond to the survey as well as site medical directors reporting their provider’s views somewhat alleviates this concern. Third, as this was a pilot phase of TD we had relatively small sample sizes for the provider surveys. That said, the goal of this analysis is to provide a framework for evaluating early implementation of TD and facilitate responsive adaptation, which requires surveying providers early in the implementation process. Fourth, we did not have socioeconomic status data on patients and did not evaluate the patient perspective of TD, which could be done in a future study. Lastly, we did not explore maintenance outcomes given the relatively recent implementation of TD at the pilot sites but plan to continue monitoring and improving these outcomes.

## Conclusions

Overall, we found that our TD virtual clinic had high implementation effectiveness and reduced patient wait-times for dermatology from > 6 months to ~ 1 week, but variable adoption across pilot clinic sites. Barriers potentially explaining the low PCP adoption included time burdens, lack of fit in clinic flow, and difficulties with dermoscopy and clinical image taking. Dermatology practices and departments implementing a new TD program may use our implementation outcomes framework for evaluation and measurement of improvement. Additionally, programs should likely prioritize facilitating interventions to alleviate barriers identified in this study prior to implementation to optimize the success of future TD programs.

## Figures and Tables

**Table 1:** Previously published teledermatology RE-AIM-based implementation outcomes, adapted from Peracca et al. 2019^13^

RE-AIM domain	Domain definition	Outcomes assessed in the current study
Patient and Provider Reach*	Degree to which patients and providers are impacted	**- Number of teledermatology patients by various characteristics** **- Percent of dermatology encounters** **- Number of completed teledermatology consults** **- Number of teledermatology consults** **- Number of providers trained**
Effectiveness and Process Measures	Ability of program to change patient-centric outcomes with quality of care	- Improvements in patient health outcomes - Diagnostic and management concordance **- Consult/appointment completion times/wait times and no-shows** - Dermatologic skill level of PCPs - Quality of life - Costs **- Patient/provider satisfaction**
Adoption	Degree to which program is used by end-users	- Stages of Implementation Completion - Understanding link between institutional readiness for change and adoption **- Percent of PCPs and dermatologists using teledermatology** - Extent to which clinics are implementing a program by understanding administrative landscape, staffing, and training needs
Implementation	Degree to which program is implemented as planned	**- Determination of detailed barriers to and facilitators of implementation** - Understanding link between individual and institutional readiness for change and successful implementation - Whether the teledermatology process is aligned with guidelines **- Assess different stakeholder perspectives**
Maintenance	Can program be sustained overtime?	- Examination of program implementation over time including assessment of long-term funding, collaboration and commitment between leadership, staff, and the community - Assessment of program responsivity such as addressing workflow and access to technology - Assess program results (e.g., change in number of teledermatology consults/encounters overtime) - Identification of training programs to ensure staff involvement and integration, and to address staff attrition

Outcomes in **bold** were considered by our study.

* While some of these outcomes may be considered Adoption outcomes, we chose to retain the original classifications in the framework published by Peracca et al.

**Table 2:** Implementation Outcomes of a Teledermatology Service 9/1/2021-4/30/2022

Outcome	Value
**Reach**	
Participating clinics, n	4
Participating attending dermatologists, n	6
Participating resident dermatologists, n	15 (total 15 residents)
Participating primary care providers, n	30
E-consults placed	218
Classified as lesion, n	154
Classified as rash, n	64
Completed TD virtual clinic visits, n	
Clinic A (urban^†^; 8 PCPs), n	20
Clinic B (urban; 16 PCPs), n	77
Clinic C (urban; 15 PCPs), n	70
Clinic D (urban; 8 PCPs), n	4
**Effectiveness**	
Video visits scheduled, % of all e-consults placed	85%
Loss-to-follow-up, % of all e-consults placed	15%
% Patient declined or unable to be reached	12%
% Other reason	3%
Completed video visits, % of scheduled video visits	92%
Video visit no-shows, % of scheduled video visits	3%
Video visit cancellations, % of scheduled video visits	5%
E-comm referrals scheduled in ≤ 3 days	80%
Mean time between e-comm referral placement and video visit, days	7.5
Average video visit length, minutes	10
Conversion to telephone visit, %	1%
Patients agree clinical goals were met, % (n = 18)	100%
Patients requiring downstream completed in-person appointments, % of TD virtual clinic patients	65%
Total downstream in-person appointments, n	111
**Adoption**	
PCPs at participating clinics that utilized TD, % of total providers*	
Clinic A (8 PCPs)	50%
Clinic B (16 PCPs)	75%
Clinic C (15 PCPs)	87%
Clinic D (8 PCPs)	13%
Percent of total dermatology referrals that utilized e-consult	
Clinic A (8 PCPs)	11%
Clinic B (16 PCPs)	19%
Clinic C (15 PCPs)	22%
Clinic D (8 PCPs)	2%

* Defined as the number of unique PCPs who placed an e-consult divided by the total number of PCPs at that clinic site.

† Urban and rural definitions based on 2010 census data and clinic county.

Abbreviations: PCP: primary care provider; TD: teledermatology

## Data Availability

Data may be provided on request to the corresponding author.

## Abbreviations

TD: teledermatology
DPC: Duke Primary Care
PCP: Primary Care Provider
APP: Advanced Practice Provider
EPIS: Exploration, Preparation, Implementation, Sustainment
EHR: Electronic Health Record
RE-AIM: Reach, Effectiveness, Adoption, Implementation, Maintenance
VA: Veteran’s Health Affairs
